# Bidirectional Associations Between Steak Doneness and Wine Co-Digestion: Protein Oxidation, Digestibility, and Phenolic Bioaccessibility During In Vitro Gastrointestinal Digestion

**DOI:** 10.3390/foods15183196

**Published:** 2026-09-10

**Authors:** Taihong Liu, Shaoyun Xing, Shuyang Sun, Chao Du, Xinye Wang, Wenguang Jiang

**Affiliations:** 1 School of Food Engineering, Ludong University, Yantai 264025, China; 18561029246@163.com (T.L.);; 2 School of Life Sciences, Ludong University, Yantai 264025, China; 3 Ningxia Changyu Longyu Estate Co. Ltd., Yinchuan 750000, China; 4 College of Enology, Northwest A & F University, Yangling 712100, China

**Keywords:** steak doneness, wine phenolics, in vitro co-digestion, protein digestion, phenolic bioaccessibility

## Abstract

Research on mixed meals using the INFOGEST protocol has often focused on individual foods, leaving co-ingested matrix associations less well characterized. To address this gap, this study investigated bidirectional associations between steaks cooked to three doneness levels (medium, SM; medium-well, SW; well-done, SD) and red wine during simulated gastrointestinal digestion, focusing on protein oxidation, apparent digestibility, secondary structure, and the apparent bioaccessibility of selected phenolic compounds. Results showed that co-digestion with red wine was associated with lower accumulation of protein oxidation markers across all doneness levels. In the gastrointestinal phase, carbonyl content was 44.5–46.8% lower and free sulfhydryl content was 53.4–59.1% higher than in steak digested alone. SW steak co-digested with wine exhibited the highest mean gastric apparent digestibility (37.7%), and FTIR indicated differences in protein secondary-structure distribution. Furthermore, mixed digests showed higher radical-scavenging activity than when wine was digested alone (an average ABTS increase of 61.4% in the gastrointestinal phase), while the steak matrix altered the digestive recovery and gastric apparent bioaccessibility of several target phenolic compounds. Overall, these findings indicate that steak doneness and red-wine co-digestion are associated with distinct matrix-dependent changes in both protein digestion and apparent phenolic bioaccessibility.

## 1. Introduction

Beef is recognized as a high-quality food, providing proteins of high biological value, B vitamins, essential minerals, and various bioactive compounds that effectively meet human nutritional requirements [1]. Steak, as a widely consumed meat dish, is typically cooked to different doneness levels, which are primarily determined by heating temperature and duration to cater to consumer preferences. Thermal processing induces oxidation in beef proteins, characterized by a decrease in sulfhydryl groups, an increase in carbonyl content, and the formation of disulfide bonds [2]. The extent of this oxidation is closely related to protein digestibility. Mild oxidation under moderate heating may partially unfold protein structures, exposing more cleavage sites for proteases and potentially enhancing digestibility. Conversely, intense heating promotes severe oxidation and aggregation, leading to compact structures that hinder enzymatic hydrolysis and reduce nutritional availability [3,4]. For instance, Chen et al. [5] suggested that a heating range of 40–85 °C was optimal for meat, beyond which oxidation became excessive. Gong, Feng, and Sun [6] found that beef processed at 85 °C exhibited high digestibility without excessive oxidation, thereby retaining nutritional quality. Additionally, the digestive process is inherently an oxidative environment that may further promote protein oxidation.

Wine, often paired with steak, is associated with health benefits when consumed in moderation [7]. These benefits are partly attributed to its high concentration of phenolic compounds, which exert potent antioxidant activity through radical scavenging, inhibition of lipid peroxidation, and metal chelation [8,9]. The INFOGEST protocol has been widely applied to characterize the digestive fate of diverse food matrices, including plant polysaccharides [10]. In simulated digestion systems, red wine has been shown to alleviate oxidative stress. For example, Van Hecke et al. [11] demonstrated that red wine, with the highest phenolic acid content, effectively reduced protein carbonylation during co-digestion with fish. Similarly, Sun et al. [7] reported that the antioxidant capacity of red wine during digestion was significantly higher than that of white wine.

Research on in vitro beef digestion has been mostly conducted in isolated, single-food systems. Even when studied within mixed meals, the focus often remains on the digestion dynamics of beef alone, while the simultaneous behavior and interactive effects of co-ingested foods are largely neglected. To address this gap, this study aimed to characterize how steak doneness relates to the co-digestion behavior of beef proteins and the red wine matrix. In such mixed matrices, digestive behaviors can differ markedly from those of steak alone because of chemical and physical associations among constituents. Important issues included how red-wine co-ingestion is associated with the oxidation and apparent digestibility of steak proteins across doneness levels, and how the apparent bioaccessibility of selected wine phenolic compounds varies with the oxidative and digestive behavior of proteins within this complex food matrix. By examining these matrix-level associations, this work provides insight into how thermal processing and food matrix effects jointly relate to digestive outcomes under controlled in vitro conditions.

## 2. Materials and Methods

### 2.1. Wine and Reagents

The wine used in this study was a red wine (Yellow Tail World Series Merlot, vintage 2020, Casella Family Brands, Yenda, NSW, Australia) with an ethanol content of 13.0% (*v*/*v*). Enzymes used in the simulated digestion, including α-amylase (EC 232-565-6), pepsin (EC 3.4.23.1), gastric lipase (EC 3.1.1.3), pancreatin (EC 3.4.21.4), bile salts, and α-glucosidase (EC 3.2.1.20), were purchased from Sigma-Aldrich (St. Louis, MO, USA). The other chemical reagents were all bought from local companies (Sinopharm Chemical Reagent Co., Ltd., Shanghai, China), and all chemicals were of sequencing grade.

### 2.2. Steak Preparation

Beef was purchased from a local market in Yantai (Shandong, China). The carcass was aged by suspension at 4 °C for 72 h post-slaughter. Subsequently, the muscle was portioned, vacuum-packaged, and stored at −20 °C until use.

Prior to cooking, samples were thawed at 4 °C overnight. The steak was uniformly sliced into 1 cm-thick portions. Cooking was performed on a preheated electric griddle (surface temperature of 200 ± 5 °C) to achieve three target doneness levels: medium (SM), medium-well (SW), and well-done (SD). The corresponding frying times for each side were 60, 75, and 90 s, respectively. The endpoint internal temperature was measured using an insulated T-type thermocouple (wire diameter 0.254 mm; Shanghai Nanpu Instrument Factory, Shanghai, China) connected to a handheld thermometer. The probe was inserted horizontally through the side of each steak, with the tip positioned at the geometric center of the thickest region, and the temperature was recorded immediately after removal from the griddle. The observed endpoint core temperatures were 61–62, 66–67, and 71–73 °C for SM, SW, and SD, respectively. After cooking, samples were cooled to room temperature. Surface moisture was gently blotted with absorbent paper, and 5 g aliquots were accurately weighed for subsequent in vitro digestion experiments.

### 2.3. Samples for in Vitro Digestive Simulation

**Figure 1:**
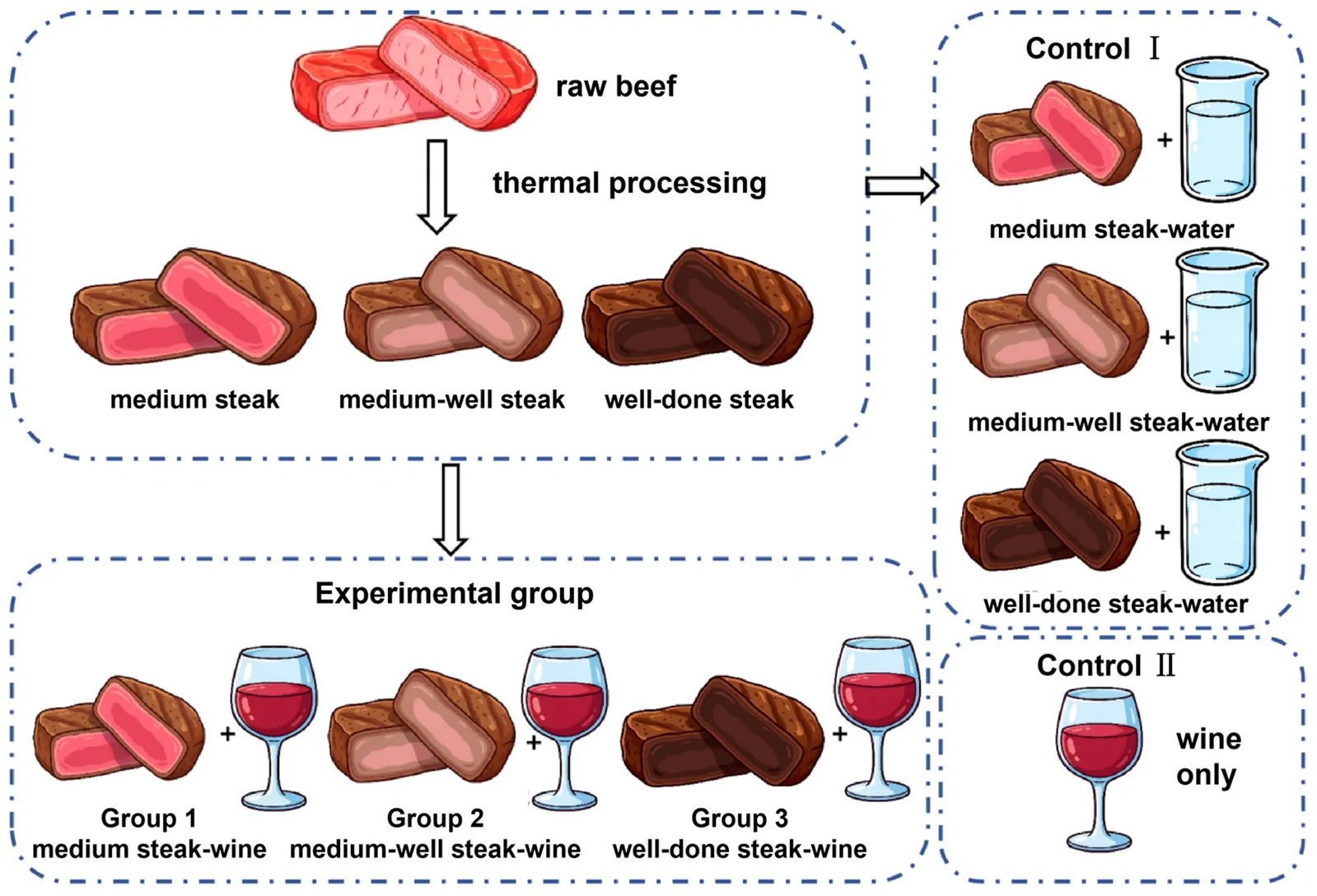
Schematic diagram of experimental design.

The experimental setup included three mixed meal samples, each consisting of 5 g of steak prepared to a specific doneness level and 10 mL of wine. Two control samples were prepared in parallel, including Control I (5 g steak and 10 mL water) and Control II (10 mL wine alone) (Figure 1). For simplicity, the mixed-meal samples were designated SMW, SWW, and SDW, corresponding to medium, medium-well, and well-done steaks with wine, respectively. The corresponding Control I samples were designated SMC, SWC, and SDC. For each doneness level, three independent steaks were used, providing three biological replicates (*n* = 3); the corresponding wine-containing and water-control digestions were prepared independently for each steak. The wine-only control was also digested in triplicate.

The INFOGEST 2.0 in vitro digestion model was used. Simulated salivary fluid, simulated gastric fluid, and simulated intestinal fluid were prepared in advance [12]. The sample was added to 15 mL of simulated salivary fluid, and pH was adjusted to 7.0 using 1 mol/L HCl, and α-amylase was added to a final concentration of 75 U/mL. Oral digestion was simulated at 37 °C for 2 min. The resulting oral-phase digest was mixed with simulated gastric fluid at a 1:1 (*v*/*v*) ratio, and the pH was adjusted to 3.0 using 1 mol/L HCl. Pepsin (2000 U/mL) and gastric lipase (60 U/mL) were added, and gastric digestion was carried out at 37 °C for 2 h. The gastric digest was adjusted to pH 6.0 using 1 mol/L NaHCO_3_, then mixed with simulated intestinal fluid at a 1:1 ratio. The pH was further adjusted to 7.0 using 1 mol/L NaOH. For the intestinal phase, pancreatin (100 U/mL) and bile salts (20 mM) were added, and digestion proceeded at 37 °C for 2 h. After digestion, the digesta from the gastric and intestinal phases were collected separately, centrifuged, and the supernatants were lyophilized and stored at −20 °C for further analysis.

### 2.4. Protein Oxidation Characterization

#### 2.4.1. Carbonyl Content

Protein carbonyl content was determined according to the DNPH derivatization method described by Han et al. [2] with slight modifications. Briefly, meat samples were homogenized in ice-cold sodium pyrophosphate buffer (pH 6.5). Proteins in the homogenate were precipitated with trichloroacetic acid (TCA, 40% *w*/*v*) and washed. The pellet was then derivatized with 2 mol/L HCl containing 10 mmol/L DNPH at 30 °C for 60 min in the dark. After stopping the reaction with TCA and subsequent washing steps to remove excess reagent, the derivatized proteins were dissolved in 6 mol/L guanidine hydrochloride (pH 2.3). The absorbance of the solution was measured at 370 nm against a reagent blank. The protein concentration of the sample was determined in parallel using the BCA assay. Carbonyl content was calculated based on the absorbance, protein concentration, and the molar absorption coefficient of the DNPH derivative (22,000 L·mol^−1^·cm^−1^), and expressed as nmol per mg protein.

#### 2.4.2. Free Sulfhydryl Content

The free sulfhydryl (SH) content was determined using DTNB reagent according to Han et al. [2]. Briefly, samples were homogenized in phosphate buffer (pH 7.0) and centrifuged. The protein concentration of the supernatant was measured using a BCA assay. An aliquot was diluted with TGE buffer (pH 8.0), mixed with DTNB reagent, and incubated at 25 °C for 60 min in the dark. Absorbance was measured at 412 nm against a blank. The SH content was calculated using the molar extinction coefficient of 13,600 L·mol^−1^·cm^−1^ for the resulting 2-nitro-5-thiobenzoate anion and expressed as nmol SH per mg protein.

### 2.5. Fourier-Transform Infrared Spectroscopy (FTIR)

The secondary structure of the proteins was determined following the method described by Qiu et al. [13]. The digested samples were initially pre-frozen at −80 °C for 1 h, subsequently freeze-dried in a lyophilizer for 48 h, and finally stored in a desiccator until analysis. Following digestion, spectral acquisition was performed using a Fourier-transform infrared spectrometer. To characterize the secondary structures of the proteins, curve fitting and double integration of the amide I band (1700–1600 cm^−1^) were conducted using OMNIC v9.8 and PeakFit v4.12 software. The spectral components corresponding to α-helix (1660–1650 cm^−1^), β-sheet (1640–1600 cm^−1^), β-turn (1700–1660 cm^−1^), and random coil (1650–1640 cm^−1^) were identified. The relative content of each secondary structure was represented by its respective peak area within this region.

### 2.6. Protein Digestibility

The protein content of the steak before digestion was determined according to Li et al. [14], and BCA-reactive protein in the total digestion mixture was measured with a BCA assay kit. Apparent protein digestibility was estimated from the decrease in BCA-reactive protein relative to the initial protein content, according to the following formulas:

Apparent digestibility (%) = (W_0_ − W_1_)/W_0_ × 100%; W_1_ = C(BCA) × V(total) × F(extraction) × F(dilution)

where W_0_ is the initial protein mass (g), W_1_ is the mass of BCA-reactive protein remaining in the total digestion mixture (g), C (BCA) is the measured protein concentration, V (total) is the total digestion volume, and F (extraction) and F (dilution) are the applicable extraction and dilution factors. Because gastric and gastrointestinal digestions were conducted as separate parallel runs, no gastric-aliquot carryover correction was applied.

### 2.7. Sodium Dodecyl Sulfate–Polyacrylamide Gel Electrophoresis (SDS-PAGE)

Protein fractions were extracted from the digestion products for electrophoretic analysis according to Li et al. [14]. Briefly, 1 mL of the digested sample was mixed with 3 mL of anhydrous ethanol and incubated at 4 °C for 12 h to precipitate proteins. After centrifugation at 10,000× *g* for 20 min at 4 °C, the obtained precipitate was resolubilized in 4 mL of extraction buffer (2% SDS in 10 mmol/L PBS, pH 7.0) and incubated at 4 °C for 12 h. The mixture was centrifuged at 1500× *g* for 15 min at 4 °C, and the resulting supernatant was collected.

The protein concentration of the supernatant was adjusted to 1.0 mg/mL. Subsequently, the sample was mixed with 4× loading buffer at a ratio of 3:1 (sample: buffer) and denatured in a water bath at 100 °C for 10 min. For electrophoresis, 10 μL of the prepared sample and 8 μL of a standard protein marker were loaded onto a 12% polyacrylamide gel. Electrophoresis was performed at a constant voltage of 90 V for 30 min, followed by 110 V for 60 min. Finally, the gel was stained with Coomassie Brilliant Blue R-250 for 45 min and destained until clear protein bands were visible.

### 2.8. Analysis of Amino Acids

The amino acid composition of the digestion products was determined using an automated amino acid analyzer (Hitachi LA8080, Hitachi High-Technologies Corporation, Tokyo, Japan) following acid hydrolysis. A 300 mg aliquot of each digested sample was mixed with 10 mL of 6 mol/L HCl. The solution was flushed with nitrogen to remove air, sealed, and hydrolyzed in an oven at 110 °C for 22 h. After hydrolysis, the solution was purged with nitrogen, filtered through a membrane, and a 1 mL aliquot of the filtrate was evaporated to dryness under a stream of nitrogen. The dried residue was redissolved in 1 mL of 20 mmol/L HCl and transferred into vials for analysis. Separation and quantification were performed using a lithium citrate buffer system with post-column ninhydrin detection. Individual amino acids were identified and quantified against external standards, and the results were expressed as mg per litre of digest (mg/L).

### 2.9. Determination of Antioxidant Activity

The antioxidant activity of the digestion samples was evaluated by measuring the radical scavenging capacity against ABTS^+^• and DPPH• radicals. The specific assay procedures were conducted according to Du et al. [15].

For the determination of ABTS radical scavenging capacity (ABTS), the ABTS^+^• solution was prepared by adding 7 mmol/L ABTS with 2.45 mmol/L potassium persulfate in the dark at room temperature for 12–16 h, then diluting with phosphate buffer (0.1 M, pH 7.4) to an absorbance of 0.70 ± 0.02 at 734 nm. For the assay, 0.1 mL of sample was mixed with 0.9 mL of ABTS^+^• solution. The mixture was vortexed and incubated in the dark at room temperature for 10 min. Absorbance was then measured at 734 nm. A blank (distilled water instead of sample) was prepared in parallel. The ABTS was calculated by the following equation:

ABTS (%) = [(A_1_ − A_2_)/A_1_] × 100%.

where A_1_ represented the blank absorbance, A_2_ represented the sample absorbance.

For the determination of DPPH radical scavenging capacity (DPPH), a 0.2 mmol/L DPPH solution was prepared in anhydrous ethanol. For the assay, 0.5 mL of sample was mixed with 1.2 mL of the DPPH solution, and the final volume was adjusted to 4.0 mL with distilled water. The mixture was vortexed and incubated in the dark at room temperature for 30 min. Absorbance was measured at 517 nm. The blank control was the absorbance of distilled water mixed with an equal volume of the sample, and the negative control was the absorbance of the sample mixed with an equal volume of absolute ethanol (as a substitute for DPPH solution). The DPPH was calculated as follows:

DPPH (%) = [A_1_ − (A_2_ − A_3_)]/A_1_ × 100%

where A_1_ represented the blank control absorbance, A_2_ represented the sample absorbance, and A_3_ represented the negative control absorbance.

### 2.10. Determination of Phenolic Compound Bioaccessibility

The bioaccessibility of phenolic acids was evaluated by analyzing their content in the digestion products. Briefly, 5 g of the digested product was mixed with 5 mL of 95% (*v*/*v*) ethanol. The mixture was vortexed vigorously and subjected to ultrasonic-assisted extraction at 40 °C for 1 h in the dark. Following extraction, the mixture was centrifuged at 16,000× *g* for 25 min at 4 °C. The supernatant was collected, filtered through a 0.22 μm membrane, and stored at −20 °C prior to HPLC analysis.

Analysis was performed on a high-performance liquid chromatography (HPLC) system equipped with a UV detector. Separation was achieved using a Besil C18-H column (250 mm × 4.6 mm, 5 μm particle size) maintained at 30 °C. The mobile phase consisted of (A) ultrapure water with 0.1% (*v*/*v*) trifluoroacetic acid and (B) a 1:1 (*v*/*v*) mixture of methanol and acetonitrile. The flow rate was 1.0 mL/min, the injection volume was 5 μL, and detection was performed at 280 nm. The gradient elution program was 0–40 min, 12–18% B; 40–45 min, 18–22% B; 45–60 min, 22–35% B; 60–65 min, 35–45% B; 65–75 min, 45–80% B; 75–80 min, 80–5% B; 80–92 min, 5% B. Phenolic acids were identified by comparing retention times with authentic standards and quantified using external calibration curves.

The apparent bioaccessibility of individual phenolic compounds was calculated as the percentage of the absolute amount recovered in the soluble digestion fraction relative to the absolute amount present in the corresponding sample before digestion, using the following formula:

Apparent bioaccessibility (%) = [C (soluble) × V (total) × F (extraction) × F (dilution)/M (initial)] × 100%

where C (soluble) is the concentration measured in the soluble digestion fraction, V (total) is the corresponding total digestion volume, F (extraction) and F (dilution) are the applicable extraction and dilution factors, and M (initial) is the amount of the target phenolic present before digestion. For co-digestion samples, M (initial) refers to the pre-digestion mixed steak–wine meal; for the wine control, it refers to the same wine dose mixed with digestive fluids.

### 2.11. Statistical Analysis

Quantitative data were expressed as the mean ± standard deviation of three biological replicates (*n* = 3). Statistical analysis was performed using IBM SPSS Statistics software (Version 23.0). Because gastric and gastrointestinal digestions were conducted as separate parallel runs, group differences were evaluated separately within each digestive phase using one-way ANOVA, followed by Duncan’s multiple range test where appropriate. For protein oxidation, apparent digestibility, and amino-acid endpoints, the steak–water and steak–wine design permits factorial evaluation of doneness, wine, and phase. Differences were considered statistically significant at *p* < 0.05. Principal component analysis (PCA) was performed in Python 3.12 using scikit-learn after z-score standardization of group-mean profiles.

## 3. Results and Discussion

### 3.1. Effect of Co-Digestion on the Digestive Behavior of Steak Proteins

#### 3.1.1. Protein Oxidation Analysis

To evaluate the association between red-wine co-ingestion and protein oxidation across different doneness levels, carbonyl and free sulfhydryl (SH) group contents were determined. Co-digestion samples from three doneness levels (SMW, SWW, and SDW) were compared with the corresponding Control I samples (SMC, SWC, and SDC) during both gastric and gastrointestinal phases.

##### Carbonyl Content

**Figure 2:**
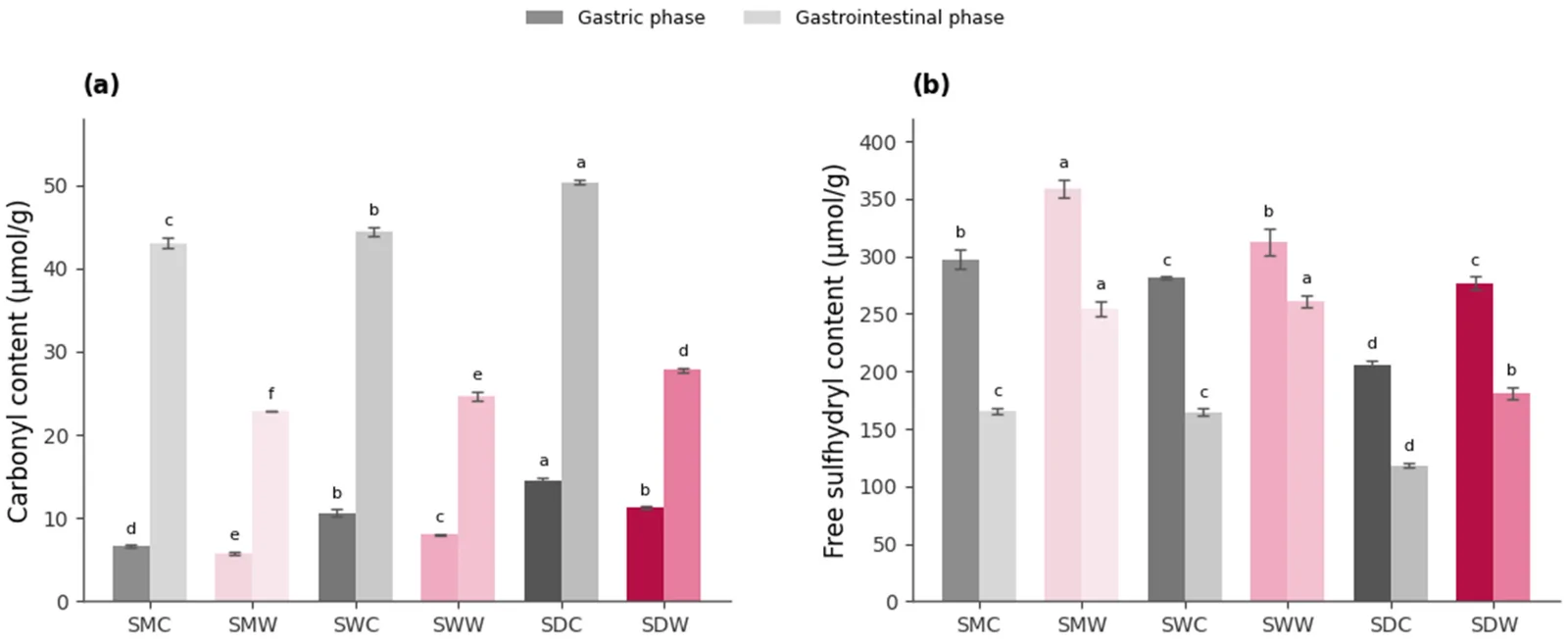
Protein carbonyl (**a**) and free sulfhydryl (**b**) contents of steak samples after simulated gastric and gastrointestinal digestion. SMC/SWC/SDC, steak digested with water (Control I); SMW/SWW/SDW, steak co-digested with wine. Mean values with different letters are significantly different within each digestive phase (*p* < 0.05). Dark-colored bars represent the gastric phase, and light-colored bars represent the gastrointestinal phase.

Protein carbonyl content, a marker of amino acid side-chain oxidation, exhibited a clear dependence on both thermal processing and digestive conditions. As shown in Figure 2a, carbonyl levels in Control I increased progressively with doneness intensity. In the gastric phase, carbonyl content rose from 6.6 µmol/g in SMC to 14.4 µmol/g in SDC. The same ordering was observed in the gastrointestinal run, where mean values were 43.0, 44.4, and 50.3 μmol/g for SMC, SWC, and SDC, respectively. These trends are consistent with findings in beef tripe, in which high-temperature cooking induced protein carbonylation and cross-linking, thereby reducing both protein digestibility and the degree of hydrolysis [16].

Co-digestion with red wine was associated with lower carbonyl accumulation across the three doneness levels. In the gastric phase, mean carbonyl content was 13.1%, 24.5%, and 22.1% lower in SMW, SWW, and SDW, respectively, than in their corresponding controls. In the gastrointestinal run, the corresponding reductions were 46.8%, 44.5%, and 44.8%. These results are consistent with attenuation of protein oxidation-marker accumulation by the red wine matrix during digestion. A similar matrix effect was reported for beef stewed with tomatoes, in which protein oxidation was significantly inhibited once the tomato proportion reached 100%, although the inhibition of protein oxidation was weaker than that of lipid oxidation [17].

The higher mean carbonyl values observed in the gastrointestinal phase compared with the gastric phase may reflect several interconnected factors. Consistent with this pattern, a survey of twelve meat-based dishes found that oxidation increased after in vitro digestion in almost all samples, with increases exceeding 6-fold in roasted pork meatballs but remaining below 60% in stewed dishes [18]. Extensive protein hydrolysis by pancreatic enzymes can yield smaller peptides and amino acids, exposing additional side-chain groups. Concurrently, oxidative reactions during digestion may modify susceptible residues, such as lysine and arginine, thereby contributing to carbonyl formation [19,20]. Furthermore, the alkaline intestinal environment may partially dissociate initially formed phenolic–protein complexes. A possible explanation is that released phenolics become less stable at higher pH and may be oxidized to quinones [21], which could react with nucleophilic amino acid residues.

##### Free Sulfhydryl Content

The content of free sulfhydryl (SH) groups, which are oxidized to form disulfide bonds, provided complementary evidence of oxidative stress and wine protection. As shown in Figure 2b, free SH content of Control I decreased significantly with increasing doneness, being lowest in SDC. In wine-co-digested samples, the free SH content of SDW was 277.8 μmol/g in the gastric phase and 180.2 μmol/g in the gastrointestinal phase. Compared to their respective Control I, wine co-digestion significantly preserved free SH groups, since in the gastric phase the increases were 20.6%, 11.1%, and 34.8% for SMW, SWW and SDW, respectively. In the gastrointestinal phase, the increases were 53.5%, 59.1%, and 53.4% for SMW, SWW and SDW, respectively.

The observed changes in free SH content reflected the combined impact of thermal processing, antioxidant protection, and digestive oxidation. Heat induced the oxidation of free sulfhydryl groups to disulfide bonds, leading to the overall reduction in SH content with increased doneness, particularly in Control I. The higher SH levels in all wine-co-digested samples are consistent with an antioxidant contribution of the red wine matrix. However, the lower SH value in the SD group, even with wine, may reflect extensive heat-induced protein aggregation that embeds sulfhydryl groups within less soluble structures; aggregation was not directly measured in this study [3].

#### 3.1.2. Secondary Structure Analysis via FTIR

To further investigate the effects of steak doneness and wine co-ingestion on protein secondary structure during simulated digestion, FTIR analysis was performed (Figure S1). All spectra exhibited a broad absorption band near 3400 cm^−1^, arising from hydroxyl and amino stretching vibrations involved in hydrogen bonding within the protein matrix. Upon co-digestion with wine, this band exhibited a consistent redshift across all doneness levels, attributable to new hydrogen bonds formed between the hydroxyl-rich wine phenolic acids and protein molecules. Meanwhile, the amide I band (1700–1600 cm^−1^), which reflects the C=O stretching vibrations of the peptide bond [13], in the control groups remained similar between SM and SW (1677.9 and 1678.1 cm^−1^) but shifted markedly to a lower wavenumber in SD (1664.6 cm^−1^), whereas the wine co-digested samples exhibited a notable blueshift relative to their controls (1681.1, 1678.9 and 1675.8 cm^−1^), most pronounced in SDW. These shifts suggest that wine phenolic acids bound to proteins through hydrogen bonds or hydrophobic interactions during digestion, thereby stabilizing the protein conformation.

**Figure 3:**
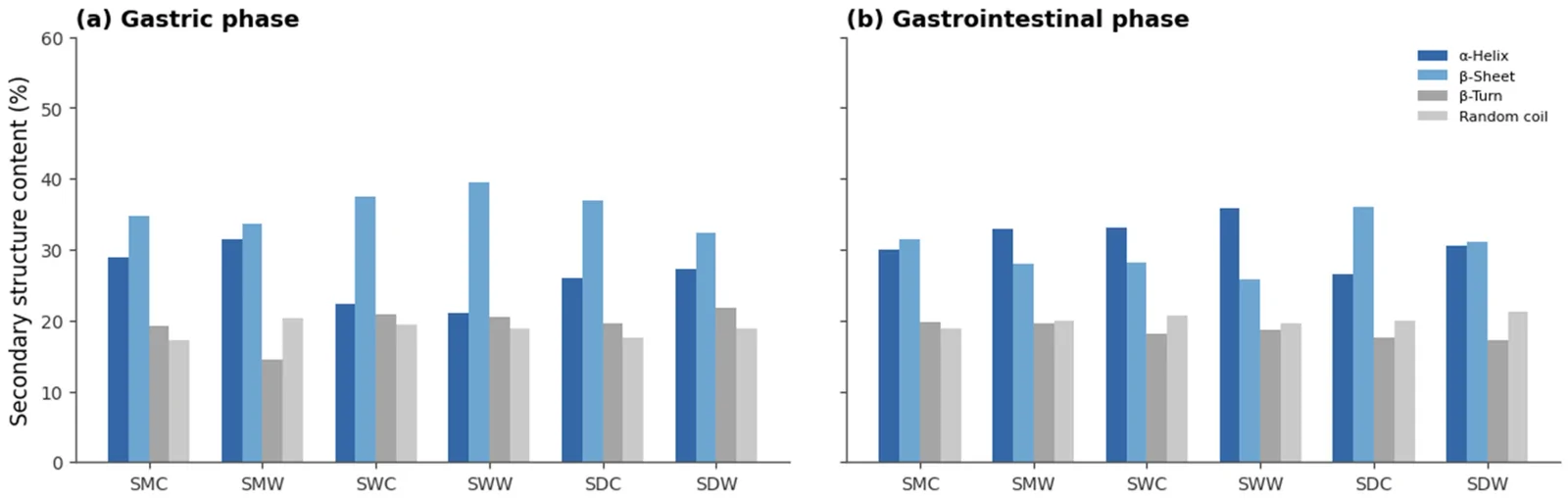
Protein secondary structure contents of steaks after simulated gastric (**a**) and gastrointestinal (**b**) digestion.

The secondary structure composition was further resolved through deconvolution and second-derivative fitting of the amide I region (Figure 3). The α-helix and β-sheet structures reflect the ordered state of protein molecules, primarily stabilized by hydrogen bonds within the peptide chain, whereas β-turn and random coil correspond to relatively loose structures. Increasing doneness was associated with a progressive decline in α-helix content and a simultaneous rise in β-sheet content. After gastrointestinal digestion, SM and SW exhibited higher α-helix and lower β-sheet content than SD across both control and experimental groups. Specifically, in the wine-co-digested groups, the α-helix content in SMW and SWW was 5.4 and 2.3 percentage points higher than in SDW, respectively, while the β-sheet content was 5.3 and 3.2 percentage points lower. The random coil content was highest in SD across both control (20.0%) and wine-co-digested (21.2%) groups, indicative of greater structural disorder.

These findings indicate that secondary-structure variations among steaks cooked to different doneness levels are closely associated with cumulative thermal exposure. In SD, prolonged heating promoted protein denaturation, with progressive α-helix unwinding and increased β-sheet and random-coil contents, consistent with the general thermal-denaturation pathway in which heating rearranges hydrogen bonds and favors aggregation-prone intermolecular β-sheet formation [5,22]. Compared with the corresponding water controls, the wine-co-digested samples exhibited a smaller extent of secondary-structure rearrangement, suggesting that the red wine matrix was associated with partial preservation of ordered protein structures during digestion. This pattern was more evident in the SMW and SWW groups, indicating that moderate thermal treatment combined with red-wine co-digestion may better preserve protein secondary structure under the present in vitro conditions [23].

#### 3.1.3. Protein Digestibility

**Figure 4:**
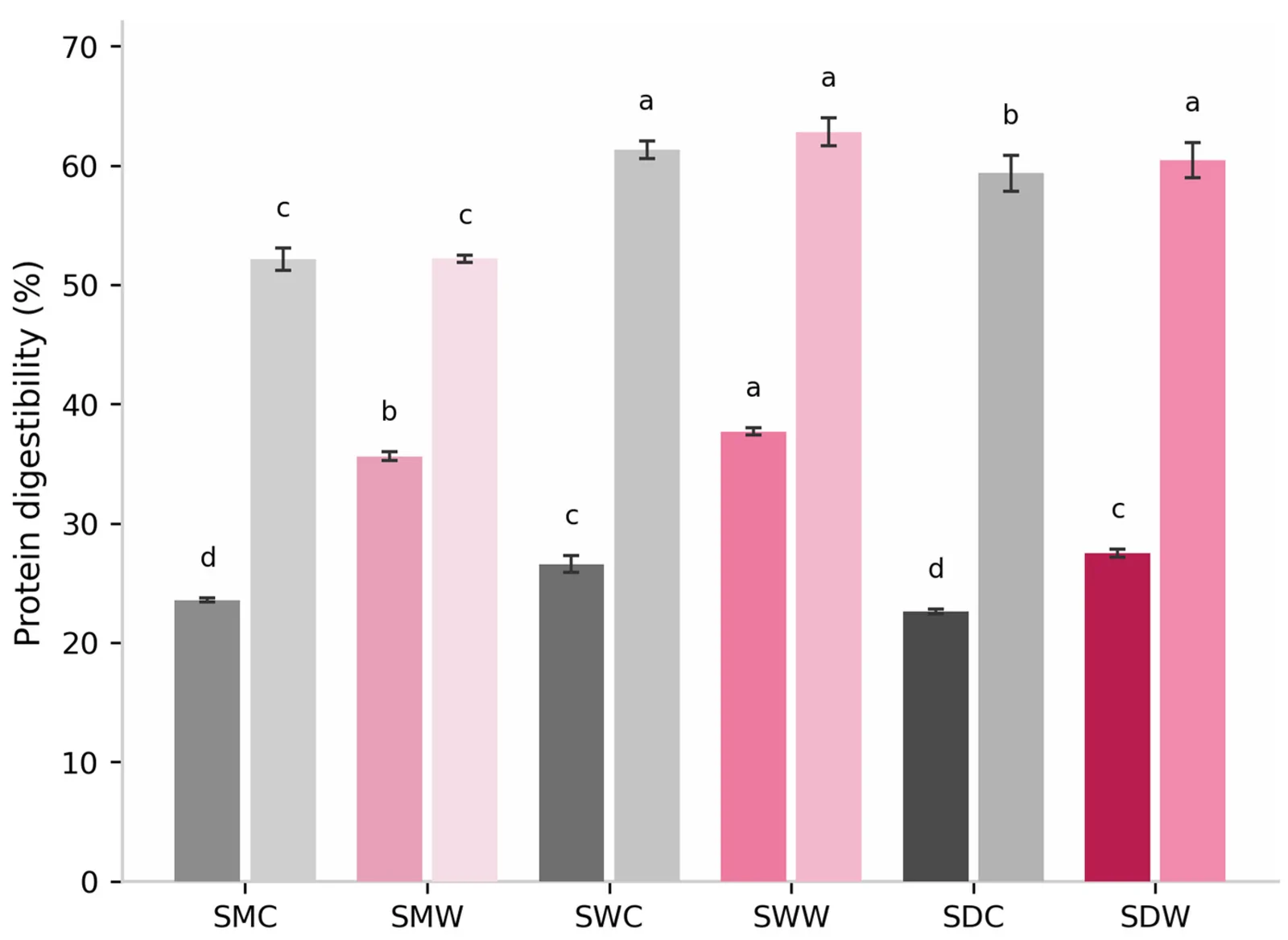
Protein digestibility of steaks after simulated gastric and gastrointestinal digestion. Data are presented as mean ± standard error. Different lowercase letters above bars indicate significant differences among samples within the same digestion phase (*p* < 0.05). Dark-colored bars represent the gastric phase, and light-colored bars represent the gastrointestinal phase.

Apparent protein digestibility, which reflects the estimated extent of protein hydrolysis based on the decrease in BCA reactive protein, is associated with potential nutrient absorption and utilization. As shown in Figure 4, gastric-phase apparent digestibility of Control I was 23.6%, 26.6%, and 22.6% for SMC, SWC, and SDC, respectively. Wine co-digestion was associated with higher gastric phase apparent digestibility across all doneness levels, resulting in increases of 12.0%, 11.1%, and 4.9% for SMW, SWW, and SDW, respectively. SWW achieved the highest mean gastric apparent digestibility (37.7%), suggesting that medium-well steak co-digested with wine had a protein conformation more susceptible to pepsin hydrolysis under the present conditions. Mean apparent digestibility values in the gastrointestinal phase were higher than those in the gastric phase. For SMW, SWW, and SDW, gastrointestinal phase values were 52.2%, 62.8%, and 60.5%, respectively, corresponding to 46.5%, 66.6%, and 119.8% higher than the gastric phase. The higher gastric digestibility of SWW is consistent with a more favorable protein conformation and potentially greater susceptibility to pepsin hydrolysis [24]. This difference may be associated with interactions between red wine components and proteins, which could promote partial protein unfolding or alter surface properties [17,25]. In that beef–tomato system, the improved digestibility was attributed to antioxidant components that limited protein oxidation, since oxidation is unfavorable for protein digestion [17]. By contrast, an opposite direction was reported when red wine was co-digested with whey proteins [26]. This discrepancy may reflect differences between a soluble dairy protein and a beef muscle matrix, as well as different wine-to-protein ratios; in both studies, however, the proteins were ultimately hydrolyzed to a similar extent by the end of the intestinal phase.

Consistent with these digestibility results, SDS-PAGE analysis (Figure S2a) showed that protein hydrolysis remained incomplete after the gastric phase, with residual bands near 80 kDa and most polypeptides distributed at 37–55 kDa. The wine co-digested samples exhibited fewer and fainter bands than their respective controls, an effect most marked for SWW, indicating more thorough gastric proteolysis in the presence of wine. After the subsequent intestinal phase (Figure S2b), the high-molecular-weight bands almost disappeared, and only a few faint bands at 25–30 kDa remained, with no significant difference among doneness levels, suggesting that proteins were ultimately hydrolyzed to a similar extent by the complete simulated gastrointestinal process.

#### 3.1.4. Amino Acid Analysis

**Figure 5:**
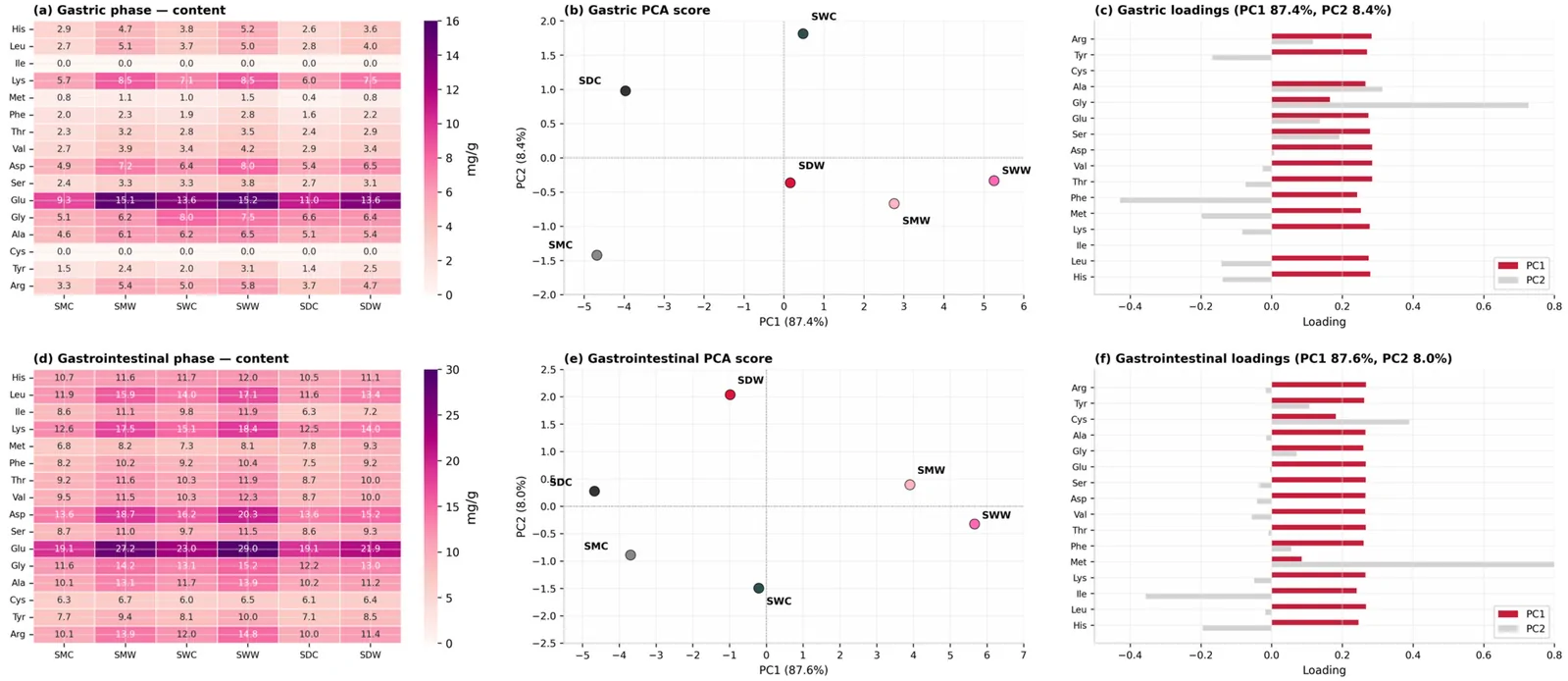
Total hydrolyzable amino acid profiles of acid-hydrolyzed digestion samples. (**a**,**d**) Heatmaps of amino acid contents (mg/g) in the gastric and gastrointestinal runs; (**b**,**e**) PCA score plots; (**c**,**f**) PC1/PC2 loading bar charts.

To characterize proteolytic products, acid-hydrolyzed digestion samples were assayed for amino acid composition in the gastric and gastrointestinal phases. A total of 16 amino acids were identified, including 8 essential amino acids (EAAs) and 8 non-essential amino acids (NEAAs) (Figure 5 and Table S1). Because samples were acid-hydrolyzed before analysis, the results represent total hydrolyzable amino acids rather than free amino acids present in the digesta.

Wine co-digestion was associated with higher EAA and NEAA contents in both digestion phases. In the gastric phase, EAAs and NEAAs increased by 29.8–51.0% and 12.3–47.8%, respectively, relative to the corresponding water controls. SWW showed the highest total hydrolyzable amino acid content (80.6 mg/g), with methionine and tyrosine showing the largest relative increases over SWC (59.2% and 54.3%, respectively). In the gastrointestinal run, total hydrolyzable amino acid contents in the wine-co-digested groups were 2.7–2.8 times those measured in the gastric run. SWW again showed the highest value (223.2 mg/g), with EAAs and NEAAs increasing by 14.2–25.8% and 11.6–30.6%, respectively, relative to the corresponding controls. Isoleucine and cysteine were detected only in the gastrointestinal phase, which may reflect phase-dependent proteolysis, peptide release, or analytical detection limits. A similar phase dependence was reported for beef tripe, where heat treatment limited amino acid release during gastric digestion and release rose rapidly in the intestinal phase under pancreatin cleavage [16].

The higher amino acid contents in the wine-co-digested groups cannot be attributed solely to enhanced steak proteolysis because the contribution of wine-derived amino acids and small peptides was not separately quantified. Red wine components may also alter protein conformation or reduce oxidative modification of susceptible residues [27]. Nevertheless, these interpretations remain literature-based hypotheses rather than experimentally demonstrated mechanisms.

In addition, PCA of z-score-standardized group-mean amino acid profiles was used to visualize differences among treatments. In the gastric phase, PC1 and PC2 explained 87.4% and 8.4% of the total variance, respectively, together accounting for 95.8%. SMC and SDC were located on the negative side of PC1, whereas SMW and SWW were positioned on the positive side; SWC and SDW were located near the PC1 origin but were separated along PC2. The loading plot indicated that PC1 primarily reflected overall amino acid abundance, whereas PC2 was mainly associated with glycine in the positive direction and phenylalanine in the negative direction.

A similar exploratory pattern was observed in the gastrointestinal phase, where PC1 and PC2 explained 87.6% and 8.0% of the total variance, respectively, together accounting for 95.6%. SMW and SWW were again positioned on the positive side of PC1, while the water controls and SDW were located on the negative side or near the PC1 origin, with additional separation along PC2. In this phase, PC2 was dominated by isoleucine in the positive direction and by methionine and cysteine in the negative direction. Collectively, these PCA patterns indicate matrix-dependent differences in amino acid profiles between wine-co-digested samples and their corresponding controls.

### 3.2. Effect of Co-Digestion on the Digestive Behavior of Wine Phenolics

To evaluate the association between steak co-ingestion and the digestive behavior of selected wine phenolics, antioxidant activity and the apparent bioaccessibility of selected phenolic compounds were assessed in the gastric and gastrointestinal runs. The tested samples included three co-digested samples (SMW, SWW, and SDW) and Control II (wine only).

#### 3.2.1. Antioxidant Activity

**Figure 6:**
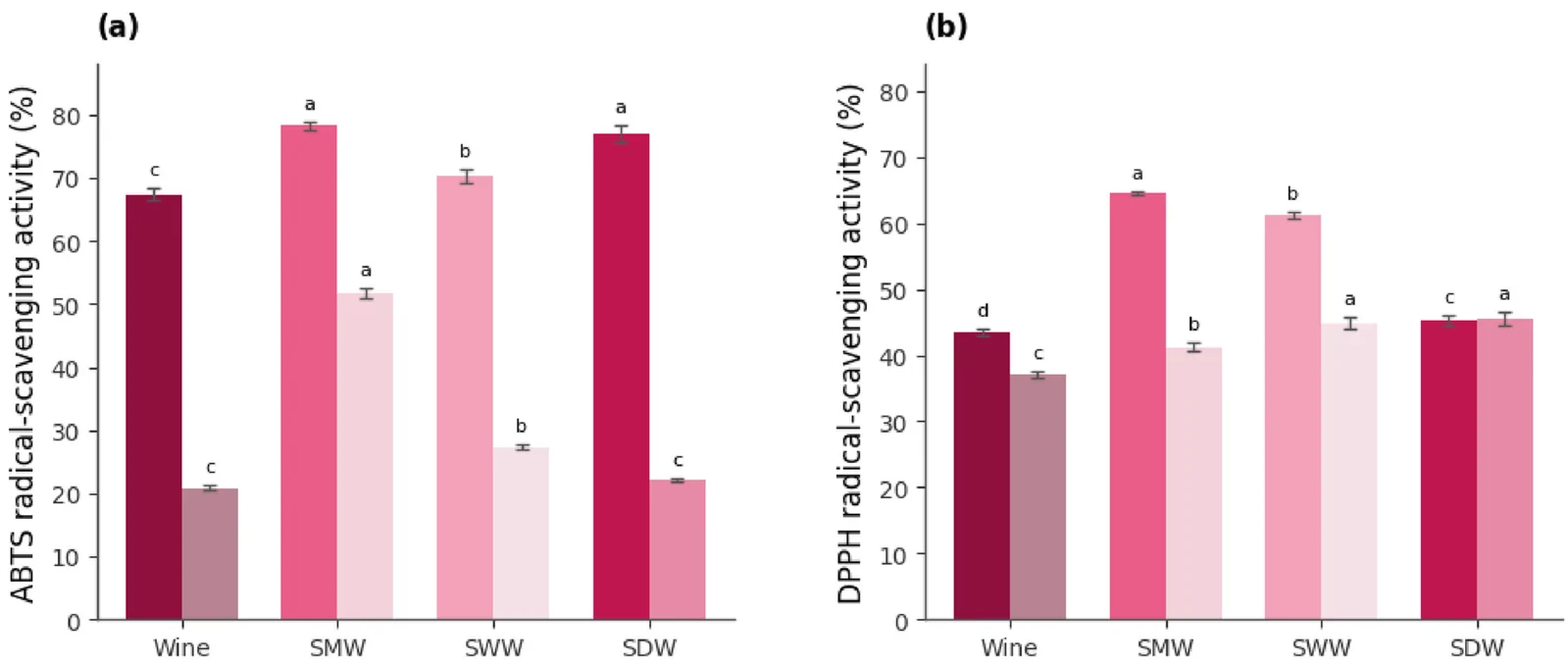
Antioxidant activity of digestion samples measured by ABTS (**a**) and DPPH (**b**) assays after simulated gastric and gastrointestinal digestion. Mean values with different letters are significantly different within each digestive phase (*p* < 0.05). Dark-colored bars represent the gastric phase, and light-colored bars represent the gastrointestinal phase.

During the gastric phase (Figure 6), the average increases across SMW, SWW, and SDW were 11.7% for ABTS and 30.9% for DPPH. In the gastrointestinal run, the corresponding increases were 61.4% for ABTS and 18.4% for DPPH. Because steak-only digests were not included in this antioxidant comparison, meat-derived peptides, amino acids, and other digestion products may also have contributed to the measured activity. The higher radical-scavenging activity of the present steak–wine co-digests relative to wine alone therefore suggests that stabilization of wine antioxidants by the digestion matrix predominated over iron-driven pro-oxidant effects under the present conditions.

Mean radical-scavenging values of the co-digested samples were lower in the gastrointestinal run than in the gastric run, with average reductions of 55.5% for ABTS and 20.7% for DPPH. This difference may be associated with pH-driven deprotonation, hydrolytic degradation, or structural rearrangement of phenolic compounds, which can alter their redox properties and reduce radical-scavenging efficiency [28]. Bile salts may further incorporate phenolics into mixed micelles or soluble complexes, limiting the accessibility of antioxidant sites, while oxidative transformation or polymerization may generate higher-molecular-weight products with different antioxidant properties [29].

#### 3.2.2. Simulated Digestion of Wine Phenolics in Gastric Phase

**Figure 7:**
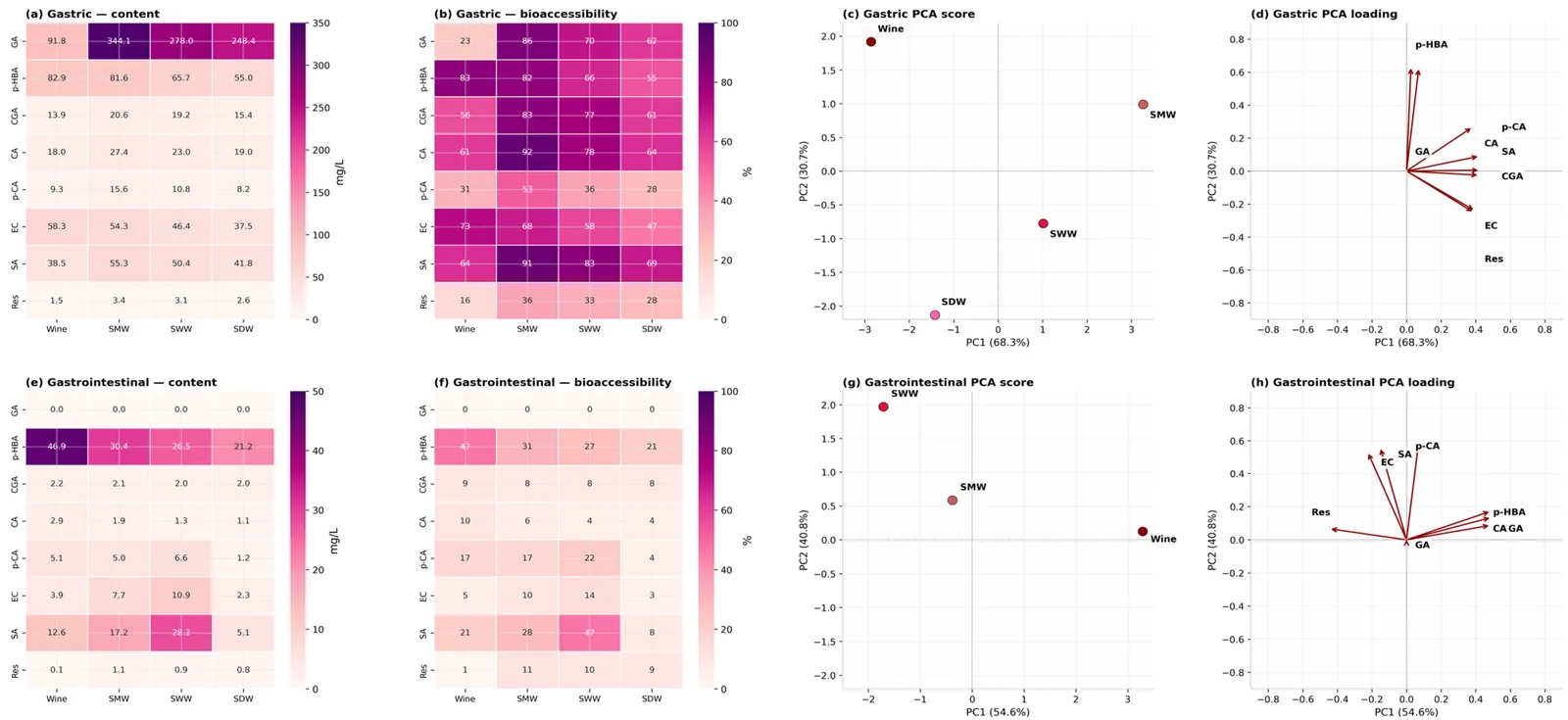
Phenolic compound profiles of digestion samples. (**a**,**e**) Concentrations (mg/L) and (**b**,**f**) apparent bioaccessibility (%) of eight phenolic compounds in the standalone gastric and separate gastrointestinal runs; (**c**,**g**) PCA score plots; (**d**,**h**) PCA loading plots.

In the gastric phase, the concentrations of most selected phenolic compounds varied among treatments (Table S2). As shown in Figure 7a, compared with Control II, co-digestion led to higher concentrations of gallic acid, caffeic acid, chlorogenic acid, syringic acid, and resveratrol. Gallic acid content was 275.0%, 203.0%, and 170.7% higher in SMW, SWW, and SDW, respectively, while resveratrol content was 122.5%, 102.5%, and 71.6% higher in the same groups. p-Coumaric acid was higher in SMW and SWW but lower in SDW. In contrast, p-hydroxybenzoic acid and epicatechin decreased. p-Hydroxybenzoic acid showed a small difference in the SM group (1.6%) but decreased by 20.7% and 33.6% in SWW and SDW, respectively. Epicatechin was 7.0%, 20.5%, and 35.8% lower in SMW, SWW, and SDW, respectively. Higher steak doneness was generally associated with lower concentrations of the measured phenolic compounds.

Apparent bioaccessibility showed similar trends (Figure 7b and Table S3). Gallic acid exhibited the largest increases over Control II (63.1%, 46.6%, and 39.2% in SMW, SWW, and SDW, respectively). Conversely, p-hydroxybenzoic acid decreased by 1.4%~28.1%, while epicatechin decreased by 5.1%~26.0%, respectively. Thus, higher doneness was generally associated with lower apparent bioaccessibility.

These trends may reflect phenolic–protein/lipid interactions and gastric-environment effects. Such interactions may protect phenolics from degradation, promote the release of free monomers from proanthocyanidins or ellagitannins [30], or improve resveratrol dissolution through association with beef lipids [31]. Conversely, the decreases in epicatechin and p-hydroxybenzoic acid may involve protein/peptide association and precipitation [32], while oxidation- and heat-induced protein restructuring may enhance phenolic entrapment [33,34], particularly in SDW. These mechanisms remain inferential because direct covalent adducts were not measured.

Furthermore, to explore differences in phenolic profiles among samples during gastric digestion, PCA was carried out as an exploratory analysis of z-score-standardized group-mean profiles (Figure 7c). The model accounted for 99.0% of the total variance, with PC1 and PC2 explaining 68.3% and 30.7%, respectively. The wine control was positioned on the negative side of PC1 and was strongly correlated with epicatechin and p-hydroxybenzoic acid. The experimental groups exhibited different distributions according to steak doneness. SMW was located on the positive side of PC1 and was associated with gallic acid, resveratrol, syringic acid, caffeic acid, and p-coumaric acid, whereas SWW and SDW shifted toward the negative PC2 direction and were separated from both the wine control and SMW.

#### 3.2.3. Simulated Digestion of Wine Phenolics in Gastrointestinal Phase

In the gastrointestinal phase, gallic acid was not detected, while the other seven phenolic compounds showed lower mean concentrations than in the gastric run. Muñoz-Bernal et al. reported a comparable decline in red wines subjected to in vitro digestion, with phenolic compounds decreasing in both type and content at later digestive stages [35]. Compared with Control II, p-hydroxybenzoic acid was 35.3%, 43.6%, and 54.8% lower in SMW, SWW, and SDW, respectively, and caffeic acid also decreased. In contrast, resveratrol concentrations were approximately 9.5~12.3 fold higher than those in Control II. p-Coumaric acid, syringic acid, and epicatechin varied non-linearly with doneness, generally increasing in SMW and SWW and peaking in SWW, where epicatechin was 182.0% higher than in Control II; however, epicatechin was 40.9% lower in SDW. Chlorogenic acid showed negligible treatment difference.

Apparent bioaccessibility followed similar trends. p-Hydroxybenzoic acid decreased from 47.2% in Control II to 30.5%, 26.7%, and 21.3% in SMW, SWW, and SDW, respectively, while caffeic acid decreased from 9.8% to 6.4%, 4.2%, and 3.6%, respectively. Conversely, resveratrol, epicatechin, syringic acid, and p-coumaric acid showed higher apparent bioaccessibility in co-digested samples, particularly SWW, where they reached 9.7%, 13.6%, 46.7%, and 22.2%, respectively [36]. These changes may reflect pH- and enzyme-dependent transformations, as well as interactions with peptides and other matrix components during gastrointestinal digestion [37,38,39]. This observation parallels the findings of Liu et al. [40], who reported that caffeic and p-coumaric acids in wine remained relatively stable during gastric digestion but underwent extensive intestinal degradation. The marked decreases in caffeic acid concentration and bioaccessibility observed in the gastrointestinal run are consistent with its documented intestinal instability [40]. However, proteolysis and bile-salt-mediated solubilization may release part of the protein-associated phenolic fraction, accounting for the higher bioaccessibility of the other compounds [27].

Moreover, PCA was applied as an exploratory analysis of z-score-standardized phenolic group-mean profiles from the gastrointestinal run (Figure 7g). PC1 and PC2 together accounted for 97.1% of the total variance, contributing 55.6% and 41.5%, respectively. The wine control was positioned on the positive side of PC1 and was associated with p-hydroxybenzoic acid, chlorogenic acid, and caffeic acid, while the three co-digested samples clustered on the negative side. SDW was located toward the negative PC2 direction and was associated with resveratrol, whereas SWW shifted toward the positive PC2 direction and was associated with syringic acid, epicatechin, and p-coumaric acid.

### 3.3. Limitations

Several limitations should be acknowledged in this study. First, only one commercial red wine was tested, without ethanol-matched, dealcoholized wine, or purified-phenolic controls; therefore, the observed effects should be attributed to the whole red wine matrix rather than specifically to its phenolic fraction. Second, steak–water HPLC, ABTS, and DPPH controls were unavailable, precluding a complete ±wine factorial analysis of phenolic and antioxidant outcomes; these results should therefore be interpreted as matrix-level associations. Finally, the sample size was limited, and the gastric and gastrointestinal digestions were conducted as separate parallel runs rather than a within-tube time course, so cross-phase differences should be interpreted cautiously. Future studies should expand the range of wine matrices, include component and matrix-matched controls, increase biological replication, and apply dynamic or time-resolved digestion models to further validate these matrix-dependent associations.

## 4. Conclusions

This in vitro study showed that steak doneness and red-wine co-ingestion were associated with different changes in both protein digestion and phenolic behavior. The wine matrix was associated with lower accumulation of protein oxidations, altered secondary-structure distribution, and higher gastric digestibility and total hydrolyzable amino acid recovery, with medium-well steak showing the most favorable protein digestion profile. For phenolics, the steak matrix enhanced radical-scavenging activity relative to wine digested alone and modified the recovery and apparent bioaccessibility of selected phenolics, although most phenolics decreased or became matrix-associated during gastrointestinal digestion. These findings highlight the importance of evaluating mixed meals rather than isolated foods, and provide a basis for optimizing meat thermal processing and beverage pairing to balance protein digestion with phenolic delivery.

## Data Availability

The original contributions presented in this study are included in the article/Supplementary Materials. Further inquiries can be directed to the corresponding authors.

## Supplementary Materials

The following supporting information can be downloaded at: https://www.mdpi.com/article/10.3390/foods15183196/s1; Figure S1: Fourier-transform infrared (FTIR) spectra of steaks after simulated gastric and gastrointestinal digestion (a) Medium; (b) Medium well; (c) Well done. Figure S2: SDS-PAGE of proteins of steak samples after simulated gastric (a) and gastrointestinal (b) digestion. Table S1: Amino acid contents (mg/g steak) in the gastric (G) and gastrointestinal (GI) digesta of steak–water and steak–wine mixtures. Table S2: Concentrations (mg/L) of phenolic compounds in the gastric and gastrointestinal digesta of the wine control and steak–wine mixtures; Table S3: Apparent bioaccessibility (%) of phenolic compounds after gastric and gastrointestinal digestion of the wine control and steak–wine mixtures.
