# Supplementary material for: Bidirectional Associations Between Steak Doneness and Wine Co-Digestion: Protein Oxidation, Digestibility, and Phenolic Bioaccessibility During In Vitro Gastrointestinal Digestion
Source: Foods. 2026 Sep 10;15(18):3196. doi: 10.3390/foods15183196 (PMC13605785; doi:10.3390/foods15183196)
Supplement: Supplementary file 1 [file foods-15-03196-s001.zip › foods-4496619-supplementary.pdf]

Supplementary Material

Figure S1. Fourier-transform infrared (FTIR) spectra of steaks after simulated gastric and gastrointestinal digestion. (a) Medium; (b) medium-well; (c) well done.

Figure S2. SDS-PAGE of proteins of steak samples after simulated gastric (a) and gastrointestinal (b) digestion.

Table S1. Amino acid contents (mg/g steak) in the gastric (G) and gastrointestinal (GI) digesta of steak–water and steak–wine mixtures.

Table S2. Concentrations (mg/L) of phenolic compounds in the gastric and gastrointestinal digesta of the wine control and steak–wine mixtures.

Table S3. Apparent bioaccessibility (%) of phenolic compounds after gastric and gastrointestinal digestion of the wine control and steak–wine mixtures.

Table S1. Amino acid contents (mg/g steak) in the gastric (G) and gastrointestinal (GI) digesta of steak–water and steak–wine mixtures.

Amino acid	Phase	SM-water	SM-wine	SW-water	SW-wine	SD-water	SD-wine
His	G	2.97 ± 0.07^{b}	4.75 ± 0.11^{d}	3.84 ± 0.13^{c}	5.17 ± 0.13^{e}	2.55 ± 0.07^{a}	3.52 ± 0.09^{c}
	GI	10.88 ± 0.28^{a}	11.63 ± 0.26^{b}	11.65 ± 0.10^{c}	12.12 ± 0.24^{c}	10.52 ± 0.04^{a}	10.96 ± 0.19^{b}
Leu	G	2.67 ± 0.03^{a}	4.96 ± 0.05^{d}	3.71 ± 0.11^{b}	5.13 ± 0.07^{d}	2.80 ± 0.02a	3.99 ± 0.04^{c}
	GI	11.90 ± 0.17^{a}	15.94 ± 0.12^{c}	13.87 ± 0.57^{b}	17.05 ± 0.09^{d}	11.66 ± 0.06^{a}	13.38 ± 0.19^{b}
Ile	G	ND	ND	ND	ND	ND	ND
	GI	8.50 ± 0.06^{c}	11.19 ± 0.40^{e}	9.79 ± 0.15^{d}	11.92 ± 0.04^{f}	6.29 ± 0.16^{a}	7.17 ± 0.14^{b}
Lys	G	5.79 ± 0.05^{a}	8.48 ± 0.22^{d}	7.07 ± 0.09^{b}	8.53 ± 0.17^{d}	5.89 ± 0.02^{a}	7.34 ± 0.07^{c}
	GI	12.71 ± 0.09^{a}	17.31 ± 0.52^{d}	15.14 ± 0.25^{c}	18.60 ± 0.12^{e}	12.52 ± 0.21^{a}	14.24 ± 0.14^{b}
Met	G	0.78 ± 0.02^{b}	1.13 ± 0.01^{d}	0.96 ± 0.02^{c}	1.53 ± 0.03^{e}	0.36 ± 0.01^{a}	0.81 ± 0.00^{b}
	GI	6.81 ± 0.12^{a}	8.21 ± 0.21^{b}	7.26 ± 0.18^{a}	8.16 ± 0.19^{b}	7.84 ± 0.07^{b}	9.38 ± 0.29^{c}
Phe	G	1.99 ± 0.05^{b}	2.35 ± 0.08^{c}	1.87 ± 0.02^{b}	2.75 ± 0.07^{d}	1.58 ± 0.00^{a}	2.17 ± 0.01^{c}
	GI	8.29 ± 0.00^{b}	10.23 ± 0.08^{d}	9.16 ± 0.19^{c}	10.60 ± 0.13^{d}	7.43 ± 0.03^{a}	9.11 ± 0.03^{c}
Thr	G	2.28 ± 0.03^{a}	3.24 ± 0.04^{c}	2.76 ± 0.06^{b}	3.52 ± 0.11^{d}	2.35 ± 0.02^{a}	2.85 ± 0.02^{b}
	GI	9.06 ± 0.27^{a}	11.42 ± 0.10^{c}	10.17 ± 0.26^{b}	12.13 ± 0.34^{c}	8.73 ± 0.11^{a}	9.81 ± 0.05^{b}
Val	G	2.73 ± 0.03^{a}	3.84 ± 0.06^{c}	3.36 ± 0.07^{b}	4.11 ± 0.04^{d}	2.86 ± 0.11^{a}	3.40 ± 0.06^{b}
	GI	9.24 ± 0.12^{b}	11.52 ± 0.17^{d}	10.34 ± 0.15^{c}	12.22 ± 0.12^{e}	8.69 ± 0.24^{a}	10.03 ± 0.27^{c}
Asp	G	4.82 ± 0.06^{a}	7.24 ± 0.08^{d}	6.40 ± 0.09^{c}	8.01 ± 0.18^{e}	5.37 ± 0.07^{b}	6.45 ± 0.12^{c}
	GI	13.62 ± 0.16^{a}	18.66 ± 0.07^{d}	16.21 ± 0.26^{c}	20.14 ± 0.26^{e}	13.37 ± 0.24^{a}	15.17 ± 0.33^{b}
Ser	G	2.41 ± 0.01^{a}	3.27 ± 0.06^{d}	3.24 ± 0.05^{c}	3.80 ± 0.04^{e}	2.75 ± 0.05^{b}	3.07 ± 0.09^{c}
	GI	8.63 ± 0.20^{a}	10.81 ± 0.05^{c}	9.78 ± 0.26^{b}	11.45 ± 0.25^{d}	8.65 ± 0.20^{a}	9.44 ± 0.08^{b}
Glu	G	9.35 ± 0.06^{a}	14.94 ± 0.13^{d}	13.58 ± 0.32^{c}	15.47 ± 0.29^{d}	11.02 ± 0.16^{b}	13.66 ± 0.44^{c}
	GI	19.13 ± 0.19^{a}	27.19 ± 0.44^{c}	23.07 ± 0.35^{b}	28.99 ± 0.50^{d}	18.93 ± 0.17^{a}	21.82 ± 0.61^{b}
Gly	G	5.05 ± 0.15^{a}	6.16 ± 0.16^{b}	8.11 ± 0.13^{d}	7.46 ± 0.12^{c}	6.61 ± 0.18^{b}	6.29 ± 0.14^{b}
	GI	11.61 ± 0.15^{a}	14.24 ± 0.39^{c}	13.08 ± 0.08^{b}	15.23 ± 0.03^{d}	12.15 ± 0.18^{a}	13.04 ± 0.52^{b}
Ala	G	4.57 ± 0.10^{a}	6.07 ± 0.14^{d}	6.10 ± 0.08^{d}	6.62 ± 0.14^{e}	5.10 ± 0.04^{b}	5.52 ± 0.12^{c}
	GI	10.12 ± 0.33^{a}	13.03 ± 0.42^{c}	11.69 ± 0.22^{b}	13.94 ± 0.27^{d}	10.15 ± 0.12^{a}	11.28 ± 0.14^{b}
Cys	G	ND	ND	ND	ND	ND	ND
	GI	6.23 ± 0.08^{a}	6.72 ± 0.06^{c}	6.12 ± 0.08^{a}	6.60 ± 0.24^{b}	6.21 ± 0.11^{a}	6.42 ± 0.03^{b}
Tyr	G	1.51 ± 0.02^{a}	2.48 ± 0.02^{c}	2.01 ± 0.06^{b}	3.11 ± 0.06^{d}	1.44 ± 0.01^{a}	2.50 ± 0.04^{c}
	GI	7.66 ± 0.23^{b}	9.33 ± 0.29^{d}	8.00 ± 0.10^{b}	10.04 ± 0.05^{e}	7.09 ± 0.14^{a}	8.45 ± 0.07^{c}
Arg	G	3.24 ± 0.07^{a}	5.29 ± 0.10^{e}	4.91 ± 0.04^{d}	5.85 ± 0.10^{f}	3.74 ± 0.05^{b}	4.69 ± 0.05^{c}
	GI	10.01 ± 0.13^{a}	13.88 ± 0.40^{c}	12.01 ± 0.43^{b}	14.89 ± 0.13^{d}	9.97 ± 0.10^{a}	11.57 ± 0.25^{b}
EAA	G	19.08 ± 0.08^{b}	28.80 ± 0.26^{e}	23.67 ± 0.20^{c}	30.73 ± 0.35^{f}	18.47 ± 0.07^{a}	24.29 ± 0.18^{d}
	GI	77.51 ± 0.07^{b}	97.51 ± 0.48^{e}	87.64 ± 0.96^{d}	101.95 ± 0.19^{f}	73.65 ± 0.34^{a}	84.10 ± 0.38^{c}
NEAA	G	30.94 ± 0.07^{a}	45.73 ± 0.37^{e}	44.38 ± 0.62^{d}	49.83 ± 0.12^{f}	36.02 ± 0.30^{b}	42.16 ± 0.58^{c}
	GI	87.40 ± 0.81^{a}	114.11 ± 0.91^{d}	99.60 ± 1.19^{c}	121.21 ± 0.87^{e}	86.92 ± 0.37^{a}	97.03 ± 0.51^{b}

Amino acid	Phase	SM-water	SM-wine	SW-water	SW-wine	SD-water	SD-wine
Total	G	50.01 ± 0.07^{a}	74.54 ± 0.41^{e}	68.06 ± 0.76^{d}	80.56 ± 0.47^{f}	54.49 ± 0.35^{b}	66.46 ± 0.65^{c}
	GI	164.91 ± 0.76^{b}	211.62 ± 1.36^{e}	187.24 ± 1.15^{d}	223.16 ± 0.69^{f}	160.57 ± 0.33^{a}	181.13 ± 0.41^{c}

Values are mean ± SD (n = 3). Different lowercase letters within a row indicate significant differences among samples within the same digestion phase ($p < 0.05$). ND, not detected. EAA, sum of essential amino acids (His, Leu, Ile, Lys, Met, Phe, Thr, Val); NEAA, sum of non-essential amino acids (Asp, Ser, Glu, Gly, Ala, Cys, Tyr, Arg); Total, sum of all quantified amino acids.

Table S2. Concentrations (mg/L) of phenolic compounds in the gastric and gastrointestinal digesta of the wine control and steak–wine mixtures.

Compound	Phase	Wine	SM-wine	SW-wine	SD-wine
Gallic acid	Gastric	93.22 ± 1.97^{a}	344.54 ± 1.90^{d}	280.21 ± 3.19^{c}	247.20 ± 6.33^{b}
	Gastrointestinal	ND	ND	ND	ND
p-Hydroxybenzoic acid	Gastric	82.29 ± 1.80^{c}	80.73 ± 1.27^{c}	65.88 ± 2.65^{b}	55.36 ± 0.64^{a}
	Gastrointestinal	47.26 ± 0.54^{d}	30.24 ± 0.^{87c}	27.08 ± 0.13^{b}	20.96 ± 0.08^{a}
Chlorogenic acid	Gastric	13.73 ± 0.26^{a}	21.15 ± 0.16^{d}	19.23 ± 0.18^{c}	15.23 ± 0.33^{b}
	Gastrointestinal	2.22 ± 0.03^{b}	2.05 ± 0.06^{a}	2.02 ± 0.03^{a}	1.97 ± 0.06^{a}
Caffeic acid	Gastric	17.90 ± 0.35^{a}	27.59 ± 0.91^{c}	22.68 ± 0.36^{b}	18.77 ± 0.46^{a}
	Gastrointestinal	2.94 ± 0.08^{d}	1.90 ± 0.04^{c}	1.26 ± 0.01^{b}	1.07 ± 0.00^{a}
p-Coumaric acid	Gastric	9.18 ± 0.10^{b}	15.74 ± 0.08^{d}	10.61 ± 0.24^{c}	8.33 ± 0.11^{a}
	Gastrointestinal	5.04 ± 0.11^{b}	5.08 ± 0.08^{b}	6.48 ± 0.07^{c}	1.21 ± 0.03^{a}
Epicatechin	Gastric	58.56 ± 0.71^{d}	54.17 ± 1.34^{c}	46.90 ± 0.67^{b}	36.64 ± 0.43^{a}
	Gastrointestinal	3.86 ± 0.04^{b}	7.73 ± 0.19^{c}	10.97 ± 0.07^{d}	2.26 ± 0.02^{a}
Syringic acid	Gastric	38.53 ± 1.49^{a}	56.10 ± 1.13^{d}	50.67 ± 0.57^{c}	42.52 ± 0.86^{b}
	Gastrointestinal	12.65 ± 0.07^{b}	17.55 ± 0.38^{c}	28.08 ± 0.74^{d}	5.08 ± 0.16^{a}
Resveratrol	Gastric	1.52 ± 0.02^{a}	3.35 ± 0.05^{d}	3.07 ± 0.02^{c}	2.57 ± 0.02^{b}
	Gastrointestinal	0.07 ± 0.00^{a}	1.05 ± 0.01^{d}	0.91 ± 0.00^{c}	0.86 ± 0.03^{b}

Values are mean ± SD (n = 3). Different lowercase letters within a row indicate significant differences among samples within the same digestion phase ($p < 0.05$). ND, not detected.

Table S3. Apparent bioaccessibility (%) of phenolic compounds after gastric and gastrointestinal digestion of the wine control and steak–wine mixtures.

Compound	Phase	Wine	SM-wine	SW-wine	SD-wine
Gallic acid	Gastric	23.3 ± 0.5^{a}	86.1 ± 0.5^{d}	70.1 ± 0.8^{c}	61.8 ± 1.6^{b}
	Gastrointestinal	ND	ND	ND	ND
p-Hydroxybenzoic acid	Gastric	82.8 ± 1.8^{c}	81.2 ± 1.3^{c}	66.3 ± 2.7^{b}	55.7 ± 0.6^{a}
	Gastrointestinal	47.5 ± 0.5^{d}	30.4 ± 0.9^{c}	27.2 ± 0.1^{b}	21.1 ± 0.1^{a}
Chlorogenic acid	Gastric	54.9 ± 1.0^{a}	84.6 ± 0.6^{d}	76.9 ± 0.7^{c}	60.9 ± 1.3^{b}
	Gastrointestinal	8.9 ± 0.1^{b}	8.2 ± 0.3^{a}	8.1 ± 0.1^{a}	7.9 ± 0.2^{a}
Caffeic acid	Gastric	60.3 ± 1.2^{a}	92.9 ± 3.1^{c}	76.3 ± 1.2^{b}	63.2 ± 1.5^{a}
	Gastrointestinal	9.9 ± 0.3^{d}	6.4 ± 0.1^{c}	4.2 ± 0.0^{b}	3.6 ± 0.0^{a}
p-Coumaric acid	Gastric	31.0 ± 0.3^{b}	53.2 ± 0.3^{d}	35.9 ± 0.8^{c}	28.2 ± 0.4^{a}
	Gastrointestinal	17.0 ± 0.4^{b}	17.2 ± 0.3^{b}	21.9 ± 0.3^{c}	4.1 ± 0.1^{a}
Epicatechin	Gastric	72.9 ± 0.9^{d}	67.4 ± 1.7^{c}	58.4 ± 0.8^{b}	45.6 ± 0.5^{a}
	Gastrointestinal	4.8 ± 0.1^{b}	9.6 ± 0.2^{c}	13.7 ± 0.1^{d}	2.8 ± 0.0^{a}
Syringic acid	Gastric	63.7 ± 2.5^{a}	92.7 ± 1.9^{d}	83.8 ± 0.9^{c}	70.3 ± 1.4^{b}
	Gastrointestinal	20.9 ± 0.1^{b}	29.0 ± 0.6^{c}	46.4 ± 1.2^{d}	8.4 ± 0.3^{a}
Resveratrol	Gastric	16.0 ± 0.2^{a}	35.3 ± 0.5^{d}	32.4 ± 0.2^{c}	27.1 ± 0.3^{b}
	Gastrointestinal	0.8 ± 0.0^{a}	11.1 ± 0.1^{d}	9.6 ± 0.0^{c}	9.0 ± 0.3^{b}

Values are mean ± SD (n = 3). Different lowercase letters within a row indicate significant differences among samples within the same digestion phase ($p < 0.05$). ND, not detected.

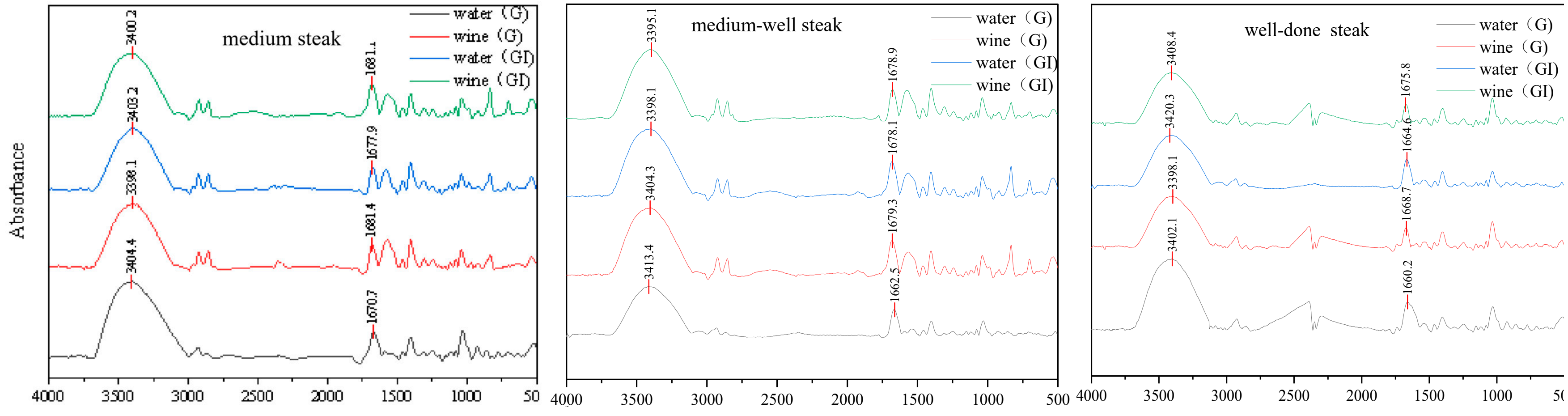

a **b** **c**

Figure S1 Fourier-transform infrared (FTIR) spectra of steaks after simulated gastric and gastrointestinal digestion. (a) Medium; (b) medium-well; (c) well done.

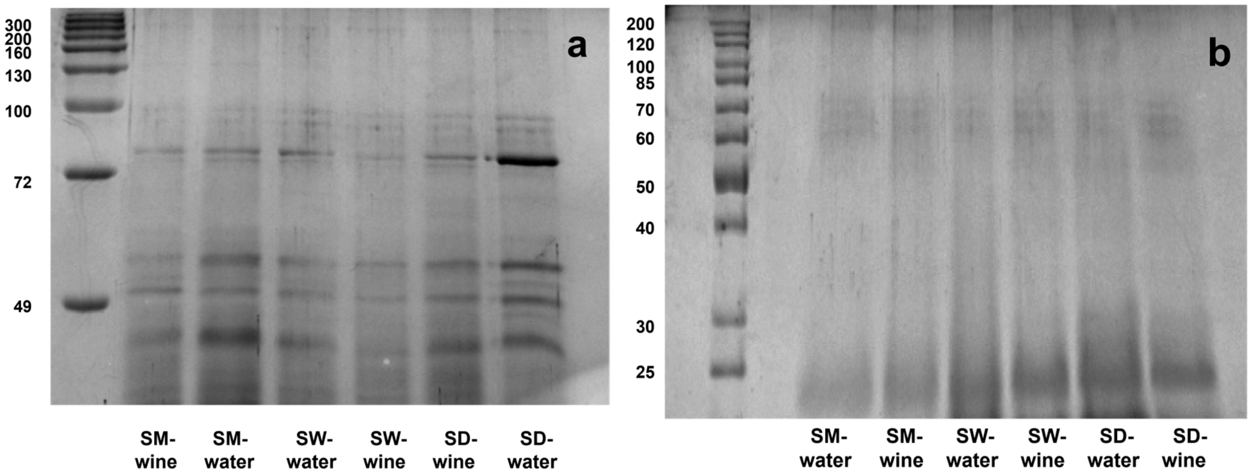

Figure S2 SDS-PAGE of proteins of steak samples after simulated gastric (a) and gastrointestinal (b) digestion.
